# Deep Learning on Point Clouds and Its Application: A Survey

**DOI:** 10.3390/s19194188

**Published:** 2019-09-26

**Authors:** Weiping Liu, Jia Sun, Wanyi Li, Ting Hu, Peng Wang

**Affiliations:** 1 School of Mathematics and Statistics, Wuhan University, Wuhan 430072, China; weipingliu_17@whu.edu.cn; 2 Institute of Automation, Chinese Academy of Sciences, Beijing 100190, China; jia.sun@ia.ac.cn (J.S.); peng_wang@ia.ac.cn (P.W.)

**Keywords:** feature learning, deep learning, point cloud, application of point cloud

## Abstract

Point cloud is a widely used 3D data form, which can be produced by depth sensors, such as Light Detection and Ranging (LIDAR) and RGB-D cameras. Being unordered and irregular, many researchers focused on the feature engineering of the point cloud. Being able to learn complex hierarchical structures, deep learning has achieved great success with images from cameras. Recently, many researchers have adapted it into the applications of the point cloud. In this paper, the recent existing point cloud feature learning methods are classified as point-based and tree-based. The former directly takes the raw point cloud as the input for deep learning. The latter first employs a k-dimensional tree (Kd-tree) structure to represent the point cloud with a regular representation and then feeds these representations into deep learning models. Their advantages and disadvantages are analyzed. The applications related to point cloud feature learning, including 3D object classification, semantic segmentation, and 3D object detection, are introduced, and the datasets and evaluation metrics are also collected. Finally, the future research trend is predicted.

## 1. Introduction

Providing detailed information for objects and environments, the point cloud is widely used in various applications such as digital preservation, reverse engineering, surveying, architecture, 3D gaming, robotics, and virtual reality. Some detailed examples are given here. In the digital preservation area, visually aesthetic and detailed 3D models of buildings and historical cities are generated by laser scanning and digital photogrammetry [1,2]. In the robotics area, point clouds are used to recognize the identity, pose, and location of the target object and obstacles for robot movement and manipulation [3,4].

Point clouds are generally produced by 3D scanners, Light Detection and Ranging (LIDAR), structure-from-motion (SFM) techniques, and recently available 3D sensors, such as Kinect and Xtion. SFM- and photogrammetry-generated point clouds usually have a low and sparse point density, while 3D scanners, LIDAR, and depth sensors can generate point clouds with more points. However, compared to the continuous surface of a 3D scene, sensed point clouds are still quite sparse. For this reason, as a pre-processing step, some techniques have been developed for densifying these point clouds, such as dense image matching. Another strategy is to use complementary data obtained from other techniques; an example is to complement data generated from structure-from-motion techniques with laser scanning. In some point clouds occlusions often occur, which request to use additional techniques for making up gaps. A common strategy in studies related to digital preservation is combining laser scanner with photogrammetry. Regarding the point density of generated point clouds, it is affected by the laser device mechanism and the object reflectivity. As an example, a typical LIDAR model, such as the HDL-64E [5], can generate a point cloud of up to ~2.2 million points per second with a range of up to 120 m. Usually, a specific device offers a user-selectable parameter range, such as rotation rate for the LIDAR sensor, to determine the density of data points. Moreover, the range accuracy of produced points can be up to ±2 cm. Point cloud consists of points with 3D unstructured vectors. Each point can be expressed by a vector, indicating its 3D coordinate and some extra feature channels, such as the intensity of reflection, color, and normals. There are three core properties for the point cloud [6], including being unordered, interaction among points, and invariance under transformations. Traditional approaches for dealing with point clouds are highly dependent on handcrafted features and well-designed optimization approaches. Features on point clouds describing their statistical properties can be divided into intrinsic or extrinsic which are invariant to several transformations [7,8]. Optimization methods are usually designed for a given application. Therefore, they have poor generalization [9,10].

Being automatically learning discriminative features, deep learning has achieved great success in object classification, semantic segmentation, object detection, etc. with optical images [11,12,13]. Recently, inspired by dense convolution, which can acquire translation invariance, feature learning approaches have been adapted to address point clouds in recent years [14,15,16,17]. These methods transform the sparse point clouds into dense tensors, including volumetric forms [18,19,20,21] and 2D images [20,22,23], or extract feature descriptors from the point clouds [24], and give these as input to deep neural networks (ConvNets). These methods usually missed much information, and the accuracy of proxy of original points became worse since they require quantization of point clouds with certain resolutions or extract descriptors from the 3D data before feeding information to ConvNets. [25] summarized the related literature and provided several directions. Different from [25], this paper focuses on methods which consume point clouds directly or convert them lossless before feature learning.

Since point clouds are important, and works of point cloud with deep learning have not been summarized yet, this paper provides an overview of the state-of-the-art progress on point clouds based on deep learning. The existing point cloud feature learning methods are classified and summarized, and their advantages and disadvantages are analyzed in this paper. Applications related to point cloud feature learning are introduced, and the related data sets and evaluation indexes are introduced. The contribution of this review has two aspects:

1. Recent advances on point clouds with deep learning are surveyed. The architectures can be classified into two categories, i.e., raw point-based and tree-based architectures. Additionally, their differences from unstructured and disordered point clouds are highlighted.
2. Applications of point clouds with deep learning are compared, and the future direction is given.

The organization of this review is as follows. The most related work of this survey is shown in Section 2. Feature learning with point clouds is introduced in Section 3, including raw point-based and tree-based types. Following this, the applications of point clouds, containing 3D object classification, semantic segmentation, and 3D object detection are described in Section 4. The performance discussion and future direction are given in Section 5. Finally, the conclusion is given in Section 6.

## 2. Related Works

Due to the availability of 3D point clouds from 3D scanners, they are widely used. Traditional methods depend on discriminative feature extractors [9,15,26]. Since deep learning has achieved great success in object classification [11,27,28,29], semantic segmentation [30,31,32], object detection [33,34,35,36,37,38,39], etc., it has been applied to address the corresponding tasks with point clouds [16,25]. The main contents of the related works are shown in Table 1.

The methods in these surveys address point clouds without raw input, missing information, or inducing heavy computing. With the emergence of PointNet, there are deep learning models taking the raw point cloud as input. Since these methods have not been surveyed yet, we will survey the recent papers in this paper.

## 3. Feature Learning on Point Cloud

**Figure 1:**
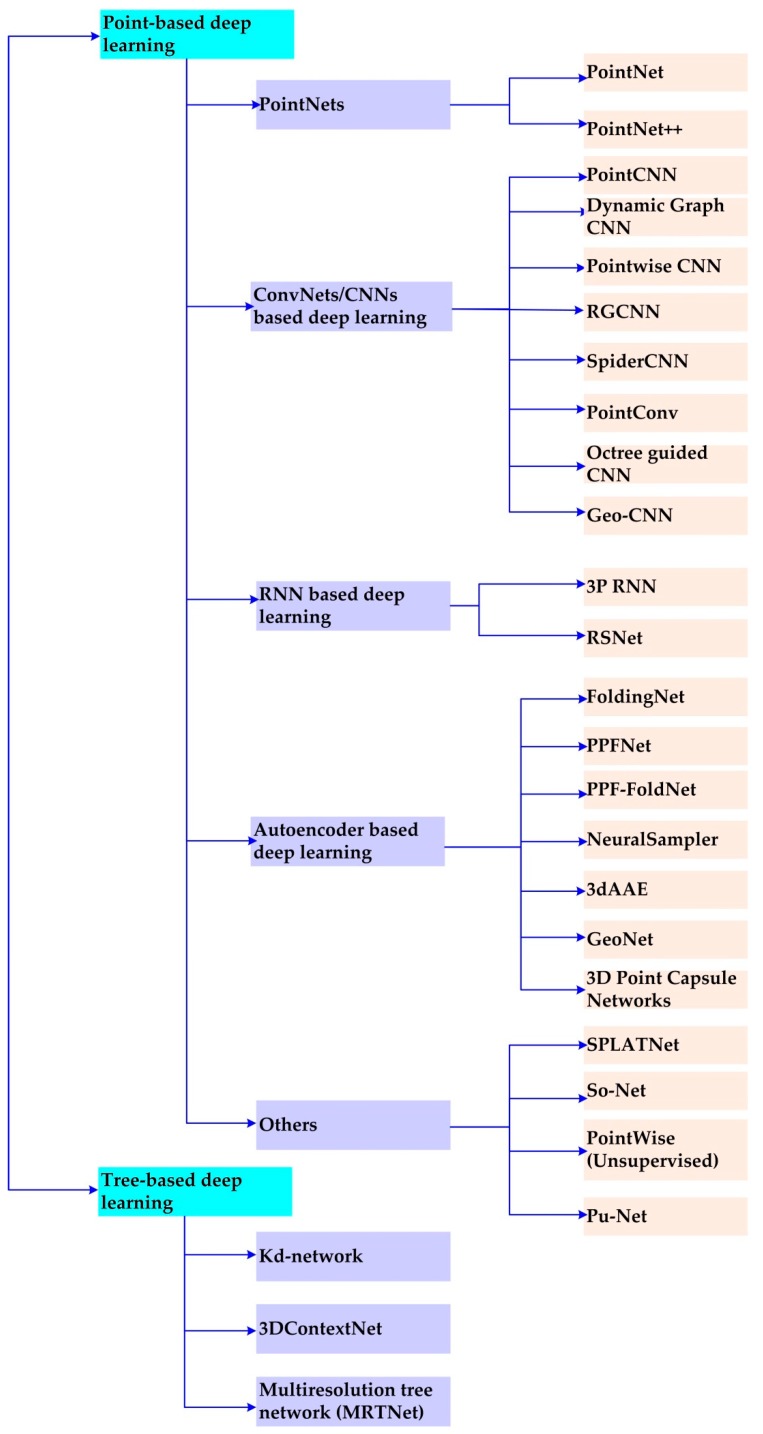
The main models for feature learning with raw point clouds as input.

At present, feature learning has been widely used with point clouds. The methods can be classified into two categories, (1) raw point-based methods, which directly consume unstructured and unordered point clouds for deep learning models and (2) k-dimensional tree (Kd-tree) based methods, which represent the point cloud regularly before feeding information into the models. Currently, there are state-of-the-art deep learning models directly addressing point clouds [6,43,44,45,46,47], and the main 18 methods are shown in Figure 1. We will first introduce the raw point-based deep learning and then the tree-based deep learning method.

### 3.1. Raw Point-Based Deep Learning

Currently, there are several models directly consuming a raw point cloud without losing information [6,43,48,49,50,51,52,53,54,55,56,57,58]. Based on the basic module of these models, they are divided into five categories, i.e., PointNet-based, deep convolutional neural networks (ConvNets)-based, recurrent neural networks (RNN)-based, autoencoder (AE)-based, and others as shown in Figure 1.

#### 3.1.1. PointNet-Based Deep Learning

There are two main architectures, including PointNet [6] and PointNet++ [43] in this section. The representative work proposed by Stanford University researchers is PointNet, which is used to directly process point clouds. Since PointNet cannot capture the local features of the point clouds, PointNet++ was then proposed. PointNet was first introduced and PointNet++ followed.

PointNet is the pioneering work with raw point clouds as input for deep learning. It has been used for 3D object detection and semantic segmentation. It was proposed to address unstructured point cloud data considering the invariance of the input point cloud arrangement. Specifically, it has two core building blocks, i.e., the transformation networks (T-Net) and the symmetric function. The former is used to align the model with the input and aggregate information from each point. It uses a spatial transformation network (STN) [59] to solve the rotation problem. STN in the computer vision community was proposed to deal with spatial invariance of objects. STN learns the rotation matrix that is most conducive to network classification or segmentation by learning the attitude information of the point cloud itself. Moreover, it employs STN twice. The first input conversion is to adjust the point cloud in the space. Intuitively, the PointNet rotates out of an angle that is more conducive to sorting or segmentation, such as turning the object to the front. The second feature transformation is to align the extracted 64-dimensional features by converting the point cloud at the feature level. Max pooling is adopted as the symmetric function for processing the point cloud. Specifically, it aggregates the high-dimensional local features of each point, which is learned from multi-layer perception (MLP) [60]. It has the capability to tackle the disorder problem and the invariance under transformations. This is because the global features of the entire point clouds can be extracted through max-pooling [12].

Since the MLP only learns the local features of each point and ignores the connections between points, PointNet fails to represent the local features of neighboring points, thus limiting its performance in complicated scenes. Based on the above analysis, PointNet cannot adequately handle local feature extraction, to address this, PointNet++ was proposed by constructing a class pyramid feature aggregation scheme. It is also used for point classification and semantic segmentation. Specifically, there are two aspects for PointNet++ to encode the local features: (1) how to divide the point cloud locally and (2) how to extract local features from the point cloud. For the first aspect, hierarchical feature learning for the point cloud is proposed. It consists of three components: the sampling layer, the grouping layer, and the PointNet layer. The sampling layer selects a series of points in the input point cloud to define the center of the local area. The sampling algorithm uses iterative farthest point sampling (FPS). Especially, FPS randomly selects a point and chooses the point furthest from the point as the starting point and then continues iteration until the desired number is selected. As for the second, PointNet++ employs PointNet to extract local features after grouping the point clouds. Therefore, the original PointNet network became a subnet in the PointNet++ network, extracting features in hierarchical iterations. Even though PointNet++ can encode the local features of the point clouds, it fails to utilize the spatial distribution of the input point cloud. This is because hierarchical feature learning fails to encode the spatial distribution in the division of the point clouds.

#### 3.1.2. ConvNets-Based Deep Learning

ConvNets is a type of feed-forward neural networks and short for deep convolutional neural networks [12,61]. Inspired by biological processes, the architecture of ConvNets is similar to the organization of the visual cortex in animals. Especially, each cortical neuron only responds to the stimuli in the receptive field. To respond to the whole field, there is overlapping area among the receptive fields in various neurons. It is always stacked with a convolution layer, rectified linear units, and pooling layers to distill features from low-level to high-level features [12,13]. ConvNets has the benefits of shared-weights, translation invariance, and feature extraction without human interference [12]. Currently, there are seven models, including Dynamic Graph convolutional neural networks (CNN) [49], PointCNN [48], regularized graph CNN (RGCNN) [50], Pointwise CNN [62], PointConv [63], Geo-CNN [64], and SpiderCNN [65], addressing the raw point cloud. These methods bring regular representation into the network before ConvNets.

Dynamic Graph CNN is a new network for classifying and dividing point cloud data and is a modification inspired by PointNet and PointNet++. PointNet only processes each point independently to achieve permutation invariance, but it ignores local features between points. To obtain the local features, Dynamic Graph CNN includes an EdgeConv layer, which solves the local feature processing problem that PointNet does not have. PointNet++ can be compared with Dynamic Graph CNN. Different from PointNet, Dynamic Graph CNN employs EdgeConv to extract features. Specifically, the EdgeConv layer is proposed to obtain local features with the tensor of N×F (N and F are the number and the dimension of the input clouds, respectively) as the input and then be applied to each given layer $\left\{a_{1,}a_{2},\ldots a_{n}\right\}$ in the MLP along the length of the output tensor to calculate the peripheral features. Finally, the merge operation is performed along adjacent peripheral features to generate a new tensor. The input includes nearby raw data $X_{i}$ and nearby K points. Specifically, each point of the original data and the attached K point will be first to generate K N×M features (M is the number of the labeled classes). Then, the N×M function will output through the pool operation.

PointCNN uses hierarchical convolution and x-Conv operators to capture local information. The benefit of x-Conv is that it considers the shapes of points without focusing on the input order of the data. It has been used in 3D object classification and semantic segmentation. Similar to the space transformation network (STN) [66], K points are taken from the data of the previous layer to predict an x-transformation matrix of K×K size (x-transformation). The features in the previous layer of the x matrix are transformed, and then the transformed features are convoluted. The convolution layer in image CNN is different from the x-Conv layer in PointCNN in only two aspects, i.e., K×K region in image CNN and K adjacent points around PointCNN representing points. In addition, the deep network assembled with the x-Conv layer is not very different from the convolution layer in the Dynamic Graph CNN. It turned out that the learning ability of the model is very strong, but the generalization is not the most advanced.

RGCNN directly consumes the point clouds with irregularity and is evaluated on point cloud segmentation. It is also accessed on point clouds with high noise. It has been used for object classification and semantic segmentation in the 3D point cloud. There are two main features of RGCNN. The first feature is that RGCNN takes the features of the points as a node on the graph based on the spectral graph theory to overcome the irregularity of the point cloud. The other feature is that it introduces the convolutional operation by Chebyshev polynomial approximation for localized filtering. As for the former method, it first collects the raw features of a point, such as color and coordinates and represents each point cloud as a vector $p_{i}$ and then feeds n points to the graph convolutional operation defined on the graph. As for the latter, it filters the nodes in the spectral domain and leverages Chebyshev approximation to dramatically decrease the computational complexity.

The new pointwise convolutional operation is proposed and then used to construct the architecture in Pointwise CNN. It is used to explore semantic segmentation and object classification in the point clouds. A pointwise convolution is introduced at each point. To implement segmentation and recognition, two pointwise convolutions are designed. The architecture of Pointwise CNN can be effective for learning local features because of the benefits of convolution operation, which uses a small kernel, such as the 3 × 3 kernel, to extract features. Unlike the traditional convolutional operation in 2D images, there is only pointwise convolution in Pointwise CNN without down-sampling or up-sampling the point clouds.

PointConv [63] is a novel convolutional operation and can be used to construct the architecture of deep convolutional neural networks addressing the irregular and unordered point clouds. It takes the coordinates of the point clouds as inputs. Especially, it is extended by the dynamic filter with non-uniform sampling. The weights in the convolution are learned by MLP, and density functions are acquired by the kernel density estimation to satisfy non-uniform sampling. This network has the scalability to deal with translation-invariant and permutation-invariant point clouds.

Inspired by the benefits of local features in the point clouds, Geo-CNN [64] aims to encode the geometric structure for a point and its corresponding neighboring point clouds through a convolutional operation. Firstly, edge features are extracted by GeoConv to encode the geometric structure with a vector and decomposed into three orthorhombic orientations. Secondly, features distilled from these directions are combined to represent the geometric structure of the point clouds with the vector and the three bases to acquire the local features.

Similar to Geo-CNN, to distill geometric features from the irregular point clouds, SpiderCNN [65] defines a novel convolutional operation. The proposed convolution is extended from the regular grids to the irregular point sets. The filter in the convolution is the product of step functions to encode the local geometric information of the point clouds. The Taylor polynomial is used to ensure the expressiveness of the SpiderCNN.

#### 3.1.3. RNN-Based Deep Learning

**Figure 2:**
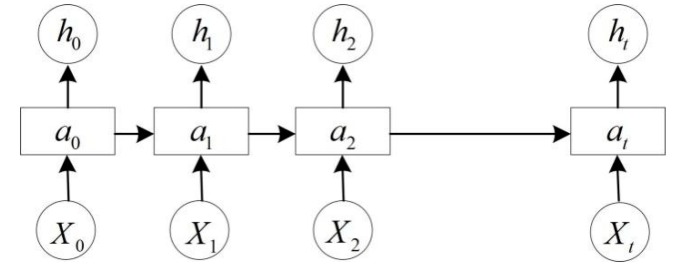
The architecture of a recurrent neural network (RNN).

A recurrent neural network (RNN) is a class of artificial neural network (ANN) where connections between nodes form a directed graph along a temporal sequence, encoding the temporal data [67]. The architecture of RNN is expressed in Figure 2, where $X_{i}\;\left(i=0,1,2,\ldots ,\;t\right)$ encodes the temporal data at the time i, and $a_{i}\;\left(i=0,1,2,\ldots ,t\right)$ are the inputs for the next time steps, while $h_{i}\;\left(i=0,1,2,\ldots ,\;t\right)$ is the output of the current time step. It is obvious that the connections between the points are a directed graph. Unlike ConvNets, RNN employs the internal states, i.e., $a_{i}\;\left(i=0,1,2,\ldots ,t\right),$ to process sequential inputs, thus making it possible to deal with the sequential tasks, such as speech recognition. It has many variations, such as Long Short-Term Memory (LSTM) [68] and bidirectional RNN [68]. Being able to capture the context, bidirectional RNN has been applied to pointwise pyramid pooling RNN (3P-RNN) [51] and recurrent slice networks (RSNets) [69] to better deal with the point clouds. 

Considering the benefits of RNN, 3P-RNN is proposed to address the semantic segmentation with raw point clouds as input. There are two main components in 3P-RNN, i.e., a pyramid pooling module and a bidirectional RNN. The former is used to extract the local spatial information, and the latter is used to acquire the global context information. 3P-RNN is inspired by PointNet as shown in Section 3.1.1. Unlike pooling in PointNet++, pointwise pyramid pooling is used to acquire the local features in 3P-RNN, which has faster speed.

RSNets is proposed to capture local structures in point clouds. The core component of the RSNets is a lightweight local dependency module. This part is the combination of the designed slice pooling layer, RNN layer, and slice unpooling layer. Specifically, the slice pooling layer is used to transform the project features of the disorder point clouds to the ordered sequence with feature vectors to be fed to the RNN layer.

#### 3.1.4. Autoencoder-Based Deep Learning

**Figure 3:**
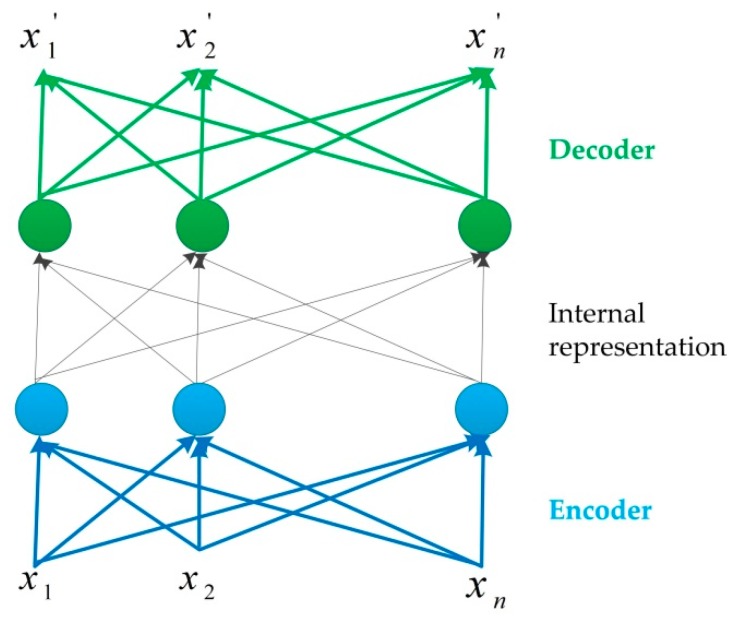
The architecture of an autoencoder.

Autoencoders (AEs) can be used to learn the representation of given data in an unsupervised manner [70] as shown in Figure 3. It is obvious that there are three stages in an autoencoder, i.e., encoder, internal representation, and decoder. Currently, it has become widely used for generative models to represent the data. It has the capability to encode the irregularity of point clouds and address the sparsity at the up-sampling stage. Researchers are beginning to employ AEs to represent them [52,54,55]. There are seven main models as shown in Figure 1, including FoldingNet, Point Pair Feature Network (PPFNet), PPF-FoldNet, NeuralSampler [55], GeoNet [71], 3D Adversarial Autoencoder (3dAAE) [72], and 3D Point-Capsule Networks.

FoldingNet is proposed to represent the point cloud from 2D to 3D with small reconstruction errors. Firstly, a graph-based encoder, combining MLP and a graph-based pooling layer, is used to acquire the local features. Secondly, a folding-based decoder is used to reconstruct the 3D point cloud from 2D images. As for the reconstruction error, the chamfer distance is used [73]. When it is used for classification, it achieves the best accuracy in the ModelNet40 dataset detailed in Section 4.1, Table 2.

The point pair feature network (PPFNet) is designed to learn globally 3D local features to discover the correspondences in unordered and sparse point clouds [53]. A novel N-tuple loss is employed to increase the intra-class difference and decrease the intra-class variations. Global information is injected into local descriptors. Integrating point pair features with normals, their corresponding 3D representations are calculated. It is designed to represent the local features of the raw point sets, which is sensitive to the global context. Inspired by PointNet, it also takes the permutation invariant network into consideration.

PPF-FoldNet was proposed to tackle the problem that PPFNet is sensitive to the rotation of the point clouds and was also used for unsupervised 3D local descriptors learning on the raw point clouds. Based on the well-known point-to-feature folding-based automatic coding, PPF-FoldNet has many desirable features: it does not require supervision or a sensitive local reference frame and can acquire rotation invariant descriptors.

NeuralSampler [55] addresses 3D point clouds of various sizes and has been used for object classification. It learns the feature representation by decoupling shape generation from surface sampling with a convolutional auto-encoder. The encoder is used to address the irregularity of the point cloud and the decoder to deal with the sparsity. Especially, a latent vector representation is calculated to encode given points, such as a surface or bounding cube.

GeoNet [71] was proposed to encode the connectivity information in the point clouds. It takes surface topology and object geometry into consideration for representing the point clouds. GeoNet employs the learned topological features for a geodesic-aware point cloud analysis. There are two components in this architecture, i.e., an autoencoder to extract a feature vector for each point and a geodesic matching (GM) layer that acts as a learned kernel function for estimating geodesic neighborhoods using the latent features.

3dAAE [72] obtains the representations of 3D shapes. It has the ability of end-to-end learning the representation of 3D point clouds. This model firstly learns a latent space for 3D shapes, and then adversarial training is used to generate the output. The authors of 3dAAE extended the autoencoder to 3D, which takes the 3D data as input and generates the corresponding 3D output.

3D Point-Capsule Networks [74] were proposed to address the sparse 3D point clouds without changing spatial arrangements. Especially, an AE is designed to do this task. This network was extended from 2D capsule networks to 3D to tackle the sparsity of the point clouds. PointNet-like input layers are employed to encode the sparsity of point clouds, and then latent capsules are used to capture information not spatially but semantically across the shape.

#### 3.1.5. Others

As stated in Section 1, there are three characteristics: unorder structure, interaction among points, and invariance under transformations. Many researchers have designed deep learning models with the raw point cloud as input. Except for the above four kinds, researchers employ special strategies to tackle the raw point dataset. For example, Self-Organizing Network (SO-Net) [57], Pointwise [58], and Pu-Net [75] use unsupervised approaches to learn the representation. SO-Net will be briefly introduced, followed by unsupervised approaches representing the point cloud.

SO-Net is a permutation-invariant network structure dealing with unordered point clouds. It utilizes the spatial distribution of the point cloud by designing a network with a constant arrangement and simulates the spatial distribution by constructing a self-organizing map (SOM) [57]. Especially, SOMs are used to acquire the hierarchical features in SO-Net. After the construction of the SOM, a feature vector is used to represent the point cloud. The point cloud automatic encoder is proposed to improve the network performance at different tasks. To maintain the order of the input point cloud, there are two core factors behind this, i.e., special network architecture and alternative SOM training. SOM does not change the topology of the input point clouds. Little information is missing before the processed point clouds feed to the network and transform the point cloud into a feature matrix, speeding up the procedure, which has tremendous advantages. There are many applications of SO-Net, including object classification, semantic segmentation, shape retrieval, etc. Due to the parallelism and simplicity of the proposed architecture, the training speed is much faster than the existing point cloud recognition network.

To calculate the hierarchical and spatial features of the point cloud, a sparse and efficient mesh filter in a lattice with high number of dimensions is proposed in Sparse Lattice Networks (SPLATNet) [56]. Similar to the architectures of ConvNets, SPLATNet makes filter neighborhoods easy to be regulated and uses hash tables to pass on only the location of the data convolved to effectively handle the sparse point cloud. It makes converting 2D points to 3D space easy and vice versa. SPLATNet uses the permutohedral lattice convolution in the Bilateral Convolution Layer, which is a generalization of bilateral filtering fusing a sparse filter into neural networks [56] to place the organization of the point cloud in each convolution operation. 

To learn the point-wise description of the point cloud, [58] uses an embedding for the cloud point through neural networks. First, an embedding space is clustered in the latent space with local structures to encode the geometric information of the point cloud. Second, the semantic point analogies are derived by computing Euclidean distance. Finally, point-correspondence is obtained by retrieving nearest-neighbors. There are two kinds of loss used in this framework, i.e., patch reconstruction loss and triplet loss. The former considers the context of the point cloud, and the latter considers that the point clouds have similar representations at the near distance and different ones at a far distance.

Pu-Net [75] is a data-driven model to learn the sparse and irregular point cloud with the raw point clouds as input. It learns the multi-level features of each point and uses the multi-branch convolution to acquire the expanded feature, which is then split to reconstruct the point cloud. There are four parts in Pu-Net, including patch extraction to acquire d point clouds with various sizes, point feature embedding to obtain the local and global geometric information of the d point clouds, feature expansion to enlarge the number of features, and coordinate reconstruction to implement the 3D coordinates of the expanded features.

Point Contextual Attention Network (PCAN) [76] is also used to encode local features. Different from PointNet++ and other neural networks, PCAN considers the task-relevant features. Especially, it first uses PointNet to extract local features and then exploits a NetVLAD layer [77] to aggregate global features. When fusing features into a discriminative global descriptor, the sampling and grouping layers in PointNet++ are first used to obtain the attention map with multi-scale contextual information, and then task-relevant features are focused.

### 3.2. Tree-Based Deep Learning

A Kd-tree is built on an eight-point point cloud. Nodes are numbered from root to leaf in the Kd-tree. Due to the irregularity of the point cloud, approaches based on a Kd-tree were proposed to explore the local and global context. Kd-tree based models take point clouds as regular presentations before feeding information into deep learning models. These methods gradually learn the representation vector of the point cloud along the tree. Experimental results on challenging datasets have shown that the Kd-tree provides distinguishing point cloud features. There are three methods, including the Kd-network [78], 3D contextual network (3DConextNet) [44], and Multiresolution Tree Networks (MRTNet) [46].

The Kd-network works with an unstructured point cloud and is designed for 3D model recognition tasks. The architecture performs a multiplication transformation and shares the parameters of these transformations according to the subdivision of the point cloud to which the Kd-tree applies. Unlike the main convolution architecture that typically requires rasterization on a uniform two- or three-dimensional grid, the Kd-network does not rely on such a mesh in any way, thus avoiding poor scaling behavior. The point layer features are hierarchically calculated at different levels in the feature learning phase. For a level, each point is processed using a shared multilayer perceptron network (MLP) as a function h in the equation. After that, a different local area representation is calculated for the same level of nodes by the corresponding function.

Just like the Kd-network, 3DContextNet was proposed to capture the local and global features of the point clouds using a Kd-tree structure. Different from the Kd-network defining operation on a Kd tree, 3DContextNet employs the Kd-tree to represent the 3D point clouds without changing the spatial relationships and can be used for 3D object classification and semantic segmentation. There are two main components in this architecture, i.e., feature learning at multi-scale and feature aggregation to extract global contextual information.

Different from Kd-network and 3DcontextNet, the point clouds are first sorted using the Kd-tree in MRTNet [46]. The Kd-tree used can represent the point clouds in a hierarchical and locality-preserving order [46]. Especially, the pooling operation defined in [46] can be used to construct the hierarchical sorting, and multiresolution scaling of the point clouds is useful for preserving the locality. Since the Kd-tree partitions the point clouds, the dependence among them is no longer kept. After the point clouds are sorted, 1D convolution and pooling are used to build the MRTNet. Experimental results on shape classification reveal the MRTNet has the benefits of small memory cost and fast convergence speed during training. MRTNet can also be used as an encoder and decoder for shape generation.

## 4. Applications of Point Clouds Using Deep Learning

There are numerous applications of the models mentioned in Section 3, which directly take the raw point cloud as input. Here, we mainly focus on three aspects, 3D object classification, semantic segmentation, and 3D object detection. First, the datasets used to evaluate the performance of the models in Section 3 are shown, and then evaluation indicators and performances of the reviewed methods regarding the three applications in each application are provided.

### 4.1. Datasets

Datasets can be divided into two categories: indoor datasets by Kinect and outdoor datasets typically obtained by 3D scanners such as LIDAR. These public datasets make it possible to compare and access various models and analyze their advantages and disadvantages. The available datasets and their descriptions and application tasks are shown in Table 2.

### 4.2. 3D Object Classification

The goal of 3D object classification is to recognize objects from a 3D point cloud [26,97,98,99], i.e., to provide a semantic object label to a separated point cloud. It has numerous applications in robotics, virtual reality, and city planning. Currently, there are several available datasets for 3D object classification in the point cloud, such as ModelNet40 and TU-Berlin as shown in Table 2. The challenges of data-related classification have three aspects, including missing data, noise, and rotation invariance.

- Missing data: Scanned models are usually occluded, and some data is lost.
- Noise: All sensors are noisy. There are different types of noise, including point perturbations and outliers. This means that a point has a certain probability within a certain radius around the location where it is sampled (disturbance), or it may appear at random locations (outliers) in space.
- Rotation invariance: Rotation and translation points should not affect classification.

Accuracy is usually used to evaluate a classification model. In general, accuracy refers to the proportion of the model that predicts the correct outcome. Formally, accuracy is defined as in Formula (1) [12]. As for the error rate, it is the misclassification rate and equal to one minus accuracy, as shown in Formula (2).

(1) $$\mathrm{accuracy}=\frac{TP+TN}{TP+TN+FP+FN}$$

(2) Error rate=1−accuracy

where TP, TN, FP, and FN are the true positive case, true negative case, false-positive case, and false-negative case, respectively.

3D object classification is receiving more and more attention and has become a very active research field. Several methods can be used for classification, such as PointNet, PointNet++, SO-Net, Dynamic Chart CNN, PointCNN, Kd-Network, 3DContextNet, Multi-Resolution Tree Network, SPLATNet, FoldingNet, and NeuralSampler. Even though there are many datasets available, the widely used datasets to access the performance of various models are ModelNet 10 and ModelNet 40. The classification performance collected from the published literatures on point cloud with these models is shown in Table 3. Class accuracy and instance accuracy are the accuracies regarding class and instance, respectively.

### 4.3. Semantic Segmentation

A point cloud is a collection of data points. It can be represented as a group, where each point can be represented by a vector, including its coordinates and additional feature channels. Once the point cloud is segmented, each segment (group) of points can be marked with a class, providing some semantics to the segment. The aim of point cloud semantic segmentation task [14,15,26,40,99] is to label each point in a point set with its corresponding semantically meaningful category.

The point cloud semantic segmentation algorithm should have three attributes:

- The segmentation algorithm should consider the specific properties of different ground objects.
- The segmentation algorithm should infer the attribute relationships of adjacent partition blocks.
- The segmentation algorithm should be applied to the point clouds acquired by different scanners.

The evaluation indicator is intersection over union (IoU) [12], a measuring accuracy of detecting corresponding objects, and is defined in Formula (3). The numerator in Formula (3) is the overlapping area between the predicted bounding box (A) and the ground-truth bounding box (B), and the denominator is the area encompassed by both A and B.

(3) $$\mathrm{IoU}=\frac{\left|A\cap B\right|}{\left|A\cap B\right|}$$

The applications of point cloud segmentation include smart vehicles, autonomous mapping, navigation, etc. There are many methods that can be used for segmentation, such as PointNet, PointNet++, SO-Net, Dynamic Graph CNN, Kd-Network, 3DContextNet, Multiresolution Tree Networks, and SPLATNet. Considering the popularity, the ShapeNet part dataset was selected to evaluate the performance of these models because many approaches exploit it. The evaluation performance for segmentation of point clouds collected from the published literatures is shown in Table 4 and Table 5 on the ShapeNet part dataset.

### 4.4. 3D Object Detection

Unlike object classification, 3D object detection in point clouds not only assigns the labels to point sets but also locates the objects of interest with bounding boxes in 3D. It becomes a challenging problem due to its discrete sampling, noise scanning, occlusion, and cluttered scenes. Compared with 3D object classification and semantic segmentation, 3D object detection with a raw point cloud is still less explored. The reasons may be the lack of large labeled point dataset. Currently, the dataset used for object detection is mainly from optical images, such as VOC2007 [101] and COCO [66]. For point clouds, the widely used dataset is KITTI [102]. Considering that only a few models consume raw point clouds directly, we provide the related works, i.e., PointRCNN [103], VoxelNet [104], MVX-Net [105], FVNet [106], F-PointNet [107], and a deep Hough voting model [108].

There are some evaluation indicators that can be used for object detection, such as Precision, Recall, $F_{1}$ score, average precision (AP), and mean average precision (mAP) as expressed by Formulas (4)–(8) [108,109,110,111,112,113,114], respectively. Precision represents the proportion of all identified correct instances. That is to say, the recall represents the proportion of all true positive examples in the sample, and these examples are correct positive examples.

In theory, the AP should be an area surrounded by a precise recall curve and two axes. This is the integral of the precision–recall curve. The AP summarizes the shape of the precision–recall curve and is defined as the mean precision at a set of equally spaced recall levels. AP measures the quality of the learning model in each category, while mAP measures the quality of the learning model in all categories. After obtaining the AP, the calculation of the mAP (the average value of all APs) becomes very simple as shown in Formula (8).

(4) $$\mathrm{Precision}=\;\frac{TP}{TP+FP}$$

(5) $$\mathrm{Recall}=\;\frac{TP}{TP+FN}$$

(6) $$F_{1}=\;\frac{2\times \mathrm{Precision}\times \mathrm{Recall}}{\mathrm{Precision}+\mathrm{Recall}}$$

(7) $$\mathrm{AP}=\;\frac{1}{N}\sum_{r}P_{\mathrm{interp}}\left({\mathrm{Recall}}^{\prime }\right)=\underset{{\mathrm{Recall}}^{\prime }>\mathrm{Recall}}{\max}P\left({\mathrm{Recall}}^{\prime }\right)$$

(8) $$\mathrm{mAP}=\frac{1}{N_{\mathrm{total}}}\sum \mathrm{AP}$$

where N is the total number of equally spaced recall levels in Formula (7), and a value of 11 is usually used for N in practice. $P_{\mathrm{interp}}$ is the precision at each recall level ${\mathrm{Recall}}^{\prime }$, and is interpolated by taking the maximum precision measured for a method for which the corresponding recall exceeds ${\mathrm{Recall}}^{\prime }$. $N_{\mathrm{total}}$ is the total number of object categories in Formula (8).

Since the dataset KITTI is a publicly available point cloud, it was used to evaluate different models. mAP is widely used to evaluate the performance of models in 3D object detection and was selected as the indicator, especially, for the dataset with only the ‘Car’ category. ScanNet and SUN RGB-D were also used. The experimental results collected from the published literatures are shown in Table 6. PointRCNN encoding the multi-scale local and rotation invariance achieves the top performance for the KITTI dataset with only the ‘Car’ category.

## 5. Discussion and Future Direction

Considering point clouds are unstructured and in disorder, especially non-Euclidean and sparse data [26], it is necessary to encode their information as completely as possible. PointNet is the first approach to deal with point clouds based on raw inputs and achieves promising results for 3D object classification and semantic segmentation. Following this, architectures from deep learning, including RNN, AE, CNN, RNN, and generative adversarial networks (GAN) [12] are introduced. Furthermore, the Kd-tree is introduced in the point clouds. Models with the raw input are surveyed, and three typical applications, including 3D object classification, semantic segmentation, and 3D object detection, are summarized. Related datasets and evaluation metrics are introduced. In this section, we will first discuss the performance, strengths, and weaknesses of the reviewed methods, and then propose some future directions.

### 5.1. Performance and Characteristics of Reviewed Methods

For 3D object classification, PointNet fails to extract the local features and only uses global features directly to obtain the probability for each class. From Table 3, we can see that the SO-Net achieves best classification performance on ModelNet 10 and ModelNet 40. The excellent performance stems from its powerful network. This may be to the special architecture of SO-Net. So-Net captures local features, global features, and a topological order of input points. Even in unsupervised learning of the point clouds, the models being able to extract local features, global features, and geometry of the point clouds have a better performance as shown in Table 3. Therefore, it is beneficial to incorporate the raw point clouds into the neural networks and also make full use of them without missing information.

For semantic segmentation, as shown in Table 4 and Table 5, it is obvious that PointNet++ and Dynamic Graph CNN achieve top performance with the mean IoU. Both PointNet++ and Dynamic Graph CNN consider the local features, which benefits the segmentation results. SPLATNet achieves about 5% higher scores over several classes, such as Knife, Ear-phone, Car, and Motor, because it employs the spatial distribution of the point clouds. Based on these analyses, integrating the local and global features extracted by deep learning models with the spatial representation of the point clouds will be useful to design a model for semantic segmentation with top performance.

For 3D object detection, as shown in Table 6, we can see that compared with other models PointRCNN can detect examples in the car class of KITTI with a higher AP. This can be attributed to its direct representation of the point cloud. It directly generates proposals from the point clouds instead of projecting them to bird’s eye view or voxels. These models show promising results for dealing with raw point clouds, encoding the point clouds that are missing little or no information.

### 5.2. Some Future Directions

From the application aspect, the models considering the spatial distribution, maintaining the topological order of input points, and extracting both global and local hierarchical features achieve the top performance. Based on those attributes contributing to a model with top performance, the further designed model should have representation power, including the spatial distribution of the whole point cloud, the topological order of input points, the global and local hierarchical features, and sparse representation. For example, one can encode the point cloud fed into the 3D neural networks. Despite much work having been done, compared with that of RGB images, the performances of methods based on point cloud processing networks for 3D object classification, semantic segmentation, and 3D object detection are still quite low. This difference due to the special inherent characteristics of the point cloud, i.e., irregular and sparse. Thus, there is still much work to conduct. Some of the aspects are stated in the following.

A promising solution is to address the raw point clouds with the ConvNets. Since ConvNets has the advantage of overlapping during convolutional operation [115,116,117], it may benefit the future architecture of deep learning models for the point cloud to take the characteristics, i.e., interaction among points, into consideration. Usually, ConvNets are used to extract multi-scale semantic features. Then, specific modules are designed for different applications. Taking semantic segmentation as an example, multi-scale features fused with skipped connections are often employed to obtain high performance, such as U-Net [31]. Recently, [118] designed a multi-resolution network for multi-scale point cloud processing and reported a 3.4% increase in IoU.

Another promising direction is to develop the architectures of the deep learning models like those in RGB images. There are many kinds of well-designed convolutional operations, such as residual module in ResNet [29] to extend the depth of the neural networks without losing accuracy, inception in GoogLeNet [27] to enlarge the width of the model with few parameters to be learned, and feature pyramid networks (FPN) [119] to extract multi-scale features. Various kinds of loss functions are also developed to train the models, such as focal loss [37] to balance the positive and negative examples and pay attention to hard examples. Since these ideas boost the application of deep models, it may be useful to design the models with the inherent characteristics of the raw point clouds in mind, such as irregular, sparse, and disorderly. For example, one can incorporate the sparse representation into the loss function to train the deep learning models for the point cloud.

Finally, zero-shot learning [115] is also an exciting topic for deep learning models directly processing raw point clouds. After obtaining the feature maps, it uses a semantic embedding for applications such as object detection. Moreover, it has the capability to recognize the unobserved class in the trained dataset. Since PointNet and EdgeConv extract global and local features of the point clouds, they can be used as feature extractors in zero-shot learning. It will facilitate learning the weights with a scarce dataset, especially in point clouds.

## 6. Conclusions

The recent existing feature learning approaches with the raw point clouds as input are classified as point-based and tree-based approaches. This survey of point cloud deep learning has a rich bibliographical content that can provide valuable insights on this important topic and encourage new research. Firstly, deep feature learning methods for raw point clouds are classified and reviewed, and the pros and cons of these methods are also analyzed. Secondly, the datasets and models with top performance regarding the applications in 3D object classification, semantic segmentation, and 3D object detection were investigated. Finally, some future directions, including model design based on ConvNets, incorporation of the inherent characteristics of point clouds with the networks, and zero-shot learning models after feature extraction by PointNet and EdgeConv, are proposed.

## Figures and Tables

**Table 1:** Related works on surveys of point clouds and their application.

Reference	Main Contents
Nygren et al. 2016 [26]	The traditional algorithms for 3D point cloud segmentation and classification
Nguyen et al. 2013 [15]	The segmentation methods for the 3D point cloud.
Ahmed et al. 2018 [16]	The 3D data from Euclidean and the non-Euclidean geometry and a discussion on how to apply deep learning to the 3D dataset.
Hana et al. 2018 [9]	The feature descriptors of point clouds with three classes, i.e., local-based, global-based, and hybrid-based.
Garcia et al. 2017 [40]	The semantic segmentation methods based on deep learning.
Bronstein et al. 2017 [41]	The problems of geometric deep learning, extending grid-like deep learning methods to non-Euclidean structures.
Griffiths et al. 2019 [42]	The classification models for processing 3D unstructured Euclidean data.

**Table 2:** Available point cloud datasets for classification, segmentation, and object detection.

Datasets Name	Descriptions	Application Tasks
ModelNet40 [18]	It consists of 12,311 CAD models in 40 object classes.	3D object classification [48,50,51,79] and shape classification [45]
ShapeNet part [80]	There are 16,881 shapes represented by 3D CAD models in 16 categories with a total of 50 parts annotated.	Part segmentation [44,48,49,56,80], shapes generation [46], and representation learning [52]
Stanford 3D semantic parsing [81]	This dataset has 271 rooms in six areas captured by 3D Matterport scanners captured by Matterport Camera.	Semantic Segmentation [8,43,44,46,48,49,78,82]
SHREC15 [18]	There are 1200 shapes in 50 categories by scanning real human participants and using 3D modeling software [79]. Each class has 24 shapes and most of these shapes are organic with different postures.	Non-rigid shape classification [43]
SHREC16 [18]	It contains about 51,300 3D models in 55 categories.	3D shape retrieval [8]
ScanNet [83]	There are 1513 scanned and reconstructed indoor scenes.	Virtual scan generation [43], segmentation [48], and classification [48]
S3DIS [81]	It consists of 271 rooms in six areas captured by 3D Matterport scanners.	3D semantic segmentation [44,48] and representation
TU-Berlin [84]	It has sketches in 250 categories. Each category has 80 sketches.	Classification [48]
ShapeNetCore [85]	It has 51,300 3D shapes in 55 categories, which is indicated by triangular meshes. The dataset is labeled manually and a subset of the ShapeNet dataset.	3D shape retrieval task [78], 3D shape retrieval task [8], and classification [8]
ModelNet10 [18]	The 10-class of Model-Net (ModelNet10) benchmarks are used for 3D shape classification. They contain 4,899 and 12,311 models respectively.	Object classification [8] Shape classification [78]
RueMonge2014 [86]	The images are multi-view in high-resolution images from a street in Paris and the number of these images is 428.	3D point cloud labeling [56]
3DMatch Benchmark [87]	It contains a total of 62 scenes.	Point Cloud representation [54]
KITTI-3D Object Detection [88,89]	There are 16 classes, including 40,000 objects in 12,000 images captured by a Velodyne laser scanner.	3D object detection [20,23,24,90]
vKITTI [91]	This dataset includes a sparse point cloud captured by LiDAR without color information. It can be used for generalization verification, but it cannot be used for supervised training.	Semantic segmentation [51]
3DRMS [92]	This dataset comes from the challenge of combining 3D and semantic information in complex scenarios and was captured by a robot that drove through a semantically rich garden with beautiful geometric details.	Semantic segmentation [51]
Cornell RGBD Dataset	It has 52 labeled point cloud indoor scenes including 24 office scenes and 28 family scenarios with the Microsoft Kinect sensor. The data set consists of approximately 550 views with 2495 segments marked with 27 object classes.	Segmentation [14]
VMR-Oakland dataset	It contains point clouds captured by mobile platforms with Navlab11 around the Carnegie Mellon University (CMU) campus.	Segmentation [14]
Robot 3D Scanning Repository	The 3D point clouds acquired by Cyberware 3030 MS are provided for both indoor and outdoor environments. Heat and color information is included in some datasets.	Segmentation [14]
ATG4D [89]	There are over 1.2 million, 5,969, and 11,969 frames in the training, validation, and test datasets, respectively. This dataset is captured by a PrimeSense sensor.	Point object detection [20]
Paris-Lille-3D [60]	There are 50 classes in 143.1M point clouds acquired by Mobile Laser Scanning.	Segmentation and classification [60]
Semantic3D [93]	There are eight classes in 1660M point clouds acquired by static LIDAR scanners.	Semantic segmentation [93]
Paris-rueMadame [94]	There are 17 classes in 20M point clouds acquired by static LIDAR.	Segmentation, classification, and detection [94]
IQmulus [61]	There are 22 classes in 12M point clouds acquired by static LIDAR.	Classification and detection [61]
MLS 1 - TUM City Campus [95,96]	There are more than 16,000 scans captured by mobile laser scanning (MLS) in this dataset.	3D detection [95,96], city modeling [95,96], and 3D change detection

**Table 3:** Classification performance on point cloud with different models.

Methods	ModelNet 10		ModelNet 40		
	Class Accuracy	Instance Accuracy	Class Accuracy	Instance Accuracy	Training
PointNet [6]	-	-	86.2	89.2	3–6 h
PointNet++ [43]	-	-	-	91.9	20 h
Deepsets [100]	-	-	-	90.0	-
SO-Net [57]	**95.5**	**95.7**	90.8	**93.4**	3 h
Dynamic Graph CNN [49]	-	-	-	92.2	-
PointCNN [48]	-	-	**91.7**	-	-
Kd-Net [78]	93.5	94.0	88.5	91.8	120 h
3DContextNet [44]	-	-	-	91.1	-
MRTNet [46]	-	-	-	91.7	-
SPLATNet [56]	-	-	83.7	86.4	-
FoldingNet [95]	-	94.4	-	88.4	-
NeuralSampler [55]	-	95.3	-	88.7	-

**Table 4:** Evaluation performance regarding for semantic segmentation on the ShapeNet part dataset [6,45,46,48,51,58,59,81].

	Intersection over Union (IoU)								
	Mean	Air- Place	Bag	Cap	Car	Chair	Ear- Phone	Guitar	Knife
PointNet [6]	83.7	83.4	78.7	82.5	74.9	89.6	73.0	91.5	85.9
PointNet++ [43]	85.1	82.4	79.0	87.7	77.3	90.8	71.8	91.0	85.9
SO-Net [57]	84.6	81.9	83.5	84.8	78.1	90.8	72.2	90.1	83.6
Dynamic Graph CNN [49]	85.1	84.2	83.7	84.4	77.1	90.9	78.5	91.5	87.3
Kd-Net [78]	82.3	80.1	74.6	74.3	70.3	88.6	73.5	90.2	87.2
3DContextNet [44]	84.3	83.3	78.0	84.2	77.2	90.1	73.1	91.6	85.9
MRTNet [46]	79.3	81.0	76.7	87.0	73.8	89.1	67.6	90.6	85.4
SPLATNet [56]	83.7	85.4	83.2	84.3	89.1	80.3	90.7	75.5	93.1

**Table 5:** Evaluation for segmentation for semantic segmentation on point cloud on ShapeNet part dataset [6,45,46,48,51,58,59,81].

	Intersection over Union (IoU)								
	Mean	Lamp	Laptop	Motor	Mug	Pistol	Rocket	Skate	Table
PointNet [6]	83.7	80.8	95.3	65.2	93.0	81.2	57.9	72.8	80.6
PointNet++ [43]	85.1	83.7	95.3	71.6	94.1	81.3	58.7	76.4	82.6
SO-Net [57]	84.6	82.3	95.2	69.3	94.2	80.0	51.6	73.1	82.6
Dynamic Graph CNN [49]	85.1	82.9	96.0	67.8	93.3	82.6	59.7	75.5	82.0
Kd-Net [78]	82.3	81.0	94.9	57.4	86.7	78.1	51.8	69.9	80.3
3DContextNet [44]	84.3	81.4	95.4	69.1	92.3	81.7	60.8	71.8	81.4
MRTNet [46]	79.3	80.6	95.1	64.4	91.8	79.7	57.0	69.1	80.6
SPLATNet [56]	83.7	83.9	96.3	75.6	95.8	83.8	64.0	75.5	81.8

**Table 6:** Point cloud object detection results [93,110]. mAP_ScanNet_, mAP_SUN RGB-D_, and mAP_3D_ results on ScanNet, SUN RGB-D, and KITTI datasets with only the ‘Car’ category.

Model	Feature Extraction	mAP_ScanNet_	mAP_SUN RGB-D_	mAP_3D_		
				Easy	Moderate	Hard
FVNet [110]	PointNet	-	-	65.43	57.34	51.85
VoxelNet [108]	-	-	-	81.97	65.46	62.85
PointRCNN [107]	PointNet++, multi-scale grouping	-	-	88.88	78.63	77.38
F-PointNet [111]	PointNet++	-	-	81.20	70.39	62.19
MVX-Net [109]	VoxelNet	-	-	83.20	72.70	65.20
Deep Hough voting model [112]	PointNet++	46.80	57.70	-	-	-
